# Optimizing bone transport strategies: a pixel value ratio-based evaluation of regeneration rates in bifocal and trifocal techniques

**DOI:** 10.3389/fsurg.2024.1494658

**Published:** 2024-12-10

**Authors:** Xin Yang, Yimurang Hamiti, Kai Liu, Sulong Wang, Xiriaili Kadier, Debin Xiong, Aihemaitijiang Yusufu

**Affiliations:** ^1^ Department of Trauma and Microreconstructive Surgery, The First Affiliated Hospital of Xinjiang Medical University, Urumqi, Xinjiang, China; ^2^ Xinjiang Key Laboratory of Trauma Repair and Reconstruction, Urumqi, Xinjiang, China

**Keywords:** bone transport, distraction osteogenesis, pixel value ratio, bifocal transport, trifocal transport, bone regeneration, Ilizarov technique

## Abstract

**Background:**

Bone transport techniques are crucial for managing large bone defects, but the optimal approach for different defect lengths remains unclear. This study aimed to compare bone regeneration rates between short bifocal bone transport (SBBT), long bifocal bone transport (LBBT), and trifocal bone transport (TBT) using pixel value ratio (PVR) as an objective quantitative measure.

**Methods:**

This retrospective study included 60 patients undergoing lower limb bone transport, divided into SBBT (*n* = 22, defects <6 cm), LBBT (*n* = 20, defects ≥6 cm), and TBT (*n* = 18, defects ≥6 cm) groups. PVR was measured at 4, 8, and 12 weeks postoperatively using standardized digital radiographs. Healing index (HI) and external fixation index (EFI) were calculated to assess treatment efficiency. Demographic data, surgical characteristics, and complications were also analyzed.

**Results:**

TBT showed significantly higher PVR values compared to LBBT at all time points (4 weeks: 0.779 ± 0.036 vs. 0.719 ± 0.027, *p* < 0.001; 8 weeks: 0.822 ± 0.027 vs. 0.787 ± 0.025, *p* = 0.008; 12 weeks: 0.866 ± 0.024 vs. 0.835 ± 0.016, *p* = 0.023) and to SBBT at 4 and 8 weeks (*p* < 0.001 and *p* = 0.016, respectively). The TBT group demonstrated significantly lower HI and EFI compared to both SBBT and LBBT groups (*p* < 0.05), indicating faster healing and shorter treatment times. Although SBBT showed slightly higher PVR values than LBBT, the differences were not statistically significant.

**Conclusion:**

Trifocal bone transport leads to faster bone regeneration and shorter treatment times compared to bifocal techniques, particularly for longer bone defects. The study demonstrates that defect length alone may not be the primary factor influencing regeneration rates in bifocal transport. PVR proves to be a reliable and cost-effective tool for assessing bone regeneration in different bone transport techniques, offering potential for guiding clinical decision-making. These findings suggest that trifocal transport should be considered as a preferred method for treating larger bone defects, especially when minimizing treatment time is crucial.

## Introduction

Bone transport techniques, based on the principle of distraction osteogenesis pioneered by Ilizarov, have revolutionized the treatment of large bone defects in orthopedic surgery (1, 2). This method harnesses the body's innate capacity for bone regeneration through the application of gradual, controlled mechanical stress (3). While bone transport has become a standard procedure for addressing bone loss due to trauma, infection, or tumor resection, optimizing the technique for different defect lengths remains a critical challenge for orthopedic surgeons (4, 5).

Traditionally, the evaluation of callus formation in the distraction gap has relied heavily on qualitative radiographic assessments, which are subject to inter-observer variability and lack objective quantification (6). This subjectivity can lead to inconsistencies in treatment decisions, potentially affecting the choice between bifocal and trifocal bone transport techniques (7). To address this issue, various quantitative methods have been proposed, including dual-energy x-ray absorptiometry (DEXA), quantitative computed tomography (QCT), and ultrasound (8, 9). However, these methods often involve additional radiation exposure, are costly, or lack practicality in routine clinical settings (10).

In recent years, the PVR has emerged as a promising quantitative tool for assessing bone regeneration during distraction osteogenesis (11, 12). PVR, calculated as the ratio of pixel values in the regenerate bone to those in adjacent normal bone on digital radiographs, provides an objective measure of bone mineralization (13). Studies have demonstrated its utility in evaluating callus maturation and guiding external fixator removal in limb lengthening procedures (14, 15). However, the application of PVR in comparing bone regeneration rates between bifocal and trifocal bone transport techniques has not been extensively investigated.

The dynamics of bone healing in bifocal vs. trifocal bone transport techniques may differ significantly, potentially influencing the interpretation of PVR values (16). Factors such as the quality of the bone ends, local blood supply, and mechanical stability may affect the mineralization process uniquely in these different approaches (17). Moreover, the impact of bone defect length on the efficacy of these techniques, as measured by PVR, remains unclear.

Understanding these nuances is crucial for developing reliable, quantitative criteria for assessing bone regeneration during bone transport and for guiding the choice between bifocal and trifocal techniques. Such criteria could significantly improve clinical decision-making, potentially leading to faster bone regeneration and reduced treatment times (18).

This study aims to investigate the utility of PVR in assessing bone regeneration during the distraction phase of bone transport, comparing bifocal bone transport for short bone defects (SBBT), bifocal bone transport for long bone defects (LBBT), and trifocal bone transport (TBT) techniques. By analyzing PVR patterns in relation to clinical outcomes and various patient factors, we seek to develop a more objective and reliable method for monitoring bone regeneration progression in different bone transport scenarios. Additionally, we aim to provide strong evidence that the trifocal bone transport technique leads to faster regeneration in the distraction gap when applied in appropriate clinical situations with suitable bone defect lengths.

Through this research, we hope to contribute to the refinement of bone transport techniques, particularly in the aspect of objectively assessing and comparing bone regeneration rates between different methods. By enhancing our ability to quantify healing outcomes, we aim to improve patient care, reduce treatment duration, and optimize the overall efficacy of bone transport procedures in orthopedic reconstruction. The findings of this study could potentially lead to more informed decision-making in selecting bone transport techniques, especially in cases where trifocal transport may offer superior regeneration rates.

By providing a quantitative comparison of regeneration rates between SBBT, LBBT, and TBT using the PVR method, this study seeks to establish an evidence-based foundation for choosing the most effective bone transport technique based on defect length. This could ultimately lead to improved clinical outcomes, shorter treatment times, and better patient satisfaction in the challenging field of limb reconstruction.

## Patients and methods

### Study design and patient selection

This study was a retrospective, single-center comparison that was conducted in January 2014 and January 2020. The Institutional Ethics Committee of our institution approved the study protocol and waived the need for participant informed consent. We included patients who had lower limb reconstruction through bone transport using the Ilizarov technique. All surgeries were performed by a team of orthopedic surgeons with extensive experience in limb reconstruction and bone transport techniques. The surgical procedures were standardized to minimize technique-related variables.

Inclusion criteria were: (1) Adult patients (≥18 years) who underwent lower limb bone transport surgery for bone defects. (2) Bone transport performed using either bifocal or trifocal technique. (3) Bone defects resulting from trauma, infection, or tumor resection. (4) Complete radiographic data available for the entire distraction phase and at least 12 months of consolidation phase. (5) Patients who complied with the prescribed post-operative protocol and follow-up schedule. Exclusion criteria included: (1) Patients under 18 years of age. (2) Incomplete radiographic data or loss to follow-up before 12 months post-surgery. (3) Patients with systemic diseases significantly affecting bone metabolism (e.g., uncontrolled diabetes, severe osteoporosis).

Based on these criteria, we initially identified 78 patients who underwent bone transport procedures during the study period. After applying the exclusion criteria, 60 patients (60 limb segments) were included in the final analysis. Some studies indicated that bone defects exceeding 6 cm were generally classified as “large segmental defects”, requiring more complex treatment strategies (19–21). Accordingly, in our study, patients were divided into three groups based on the bone transport technique and the length of bone defect: 1. Short Bifocal Bone Transport (SBBT, *n* = 22): bone defects of <6 cm treated with bifocal transport. 2. Long Bifocal Bone Transport (LBBT, *n* = 20): bone defects of ≥6 cm treated with bifocal transport. 3. Trifocal Bone Transport (TBT, *n* = 18): bone defects of ≥6 cm treated with trifocal transport.

### Surgical technique and postoperative management

All surgeries were performed by a team of experienced orthopedic surgeons using a standardized technique. Under general anesthesia, an external fixator was applied. Osteotomy was performed using a low-energy technique to preserve periosteal blood supply. For SBBT and LBBT, a single osteotomy was performed, while TBT involved two osteotomies.

Postoperatively, patients received prophylactic antibiotics and thromboprophylaxis according to our institution's protocol. Pin site care was initiated on the second postoperative day. After a latency period of 7 days, distraction was started at a rate of 1 mm per day, divided into four increments. The distraction rate was adjusted based on the quality of regenerate formation as assessed on follow-up radiographs. Patients were encouraged to perform early joint mobilization and partial weight-bearing as tolerated. Regular follow-ups were scheduled weekly during the distraction phase and monthly during the consolidation phase.

### Radiographic evaluation and PVR measurement

**Figure 1:**
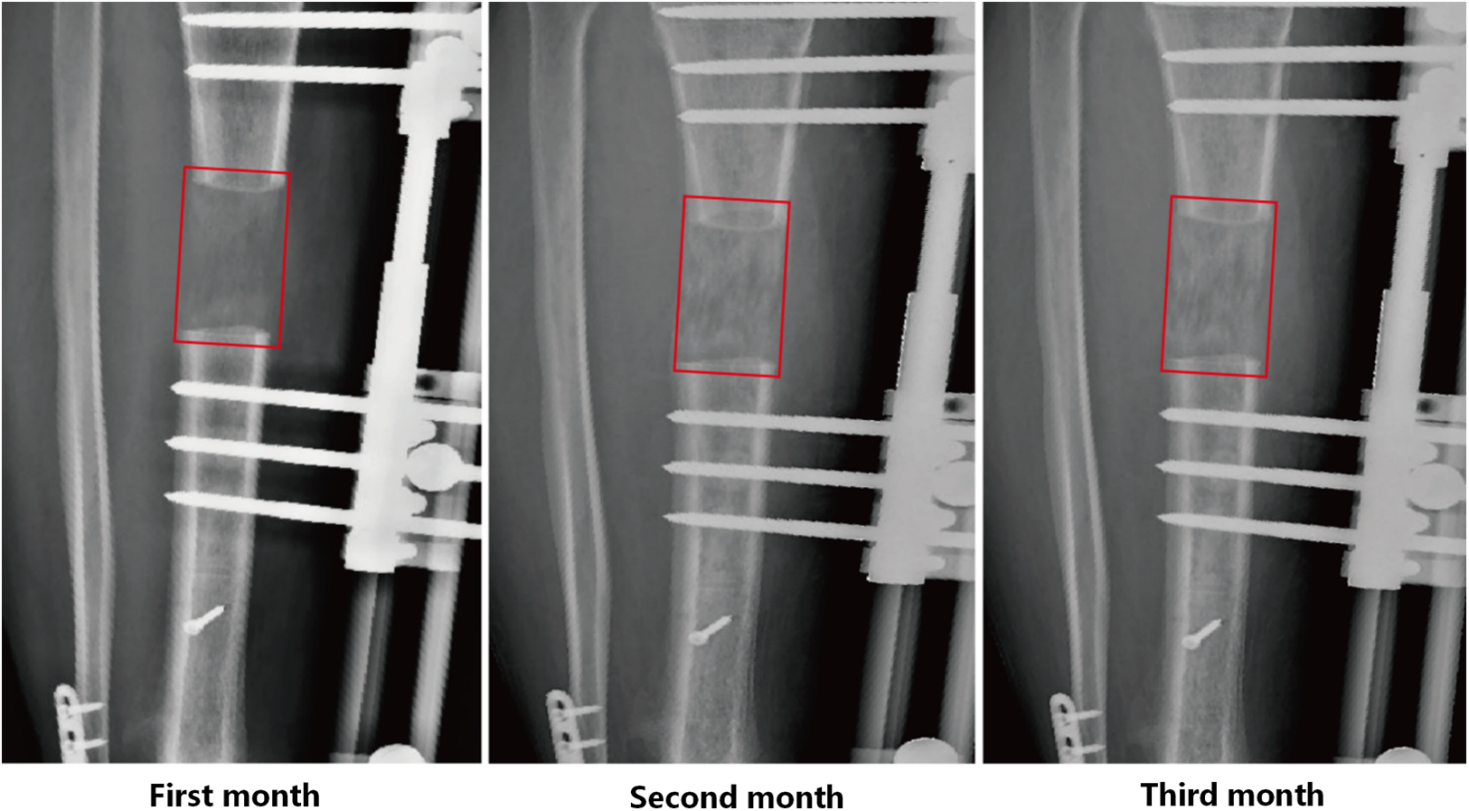
Radiographic assessment of bone regeneration during bone transport.

All radiographs were obtained through the Picture Archiving and Communication System (PACS) (Carestream Vue PACS, Rochester, NY, USA). Our radiographs were taken by professional and designated technicians using the MobiEye 700 (Mindray, China). This advanced mobile digital x-ray machine is equipped with an innovative bionic arm, which endows it with remarkable flexibility and a wide scope of movement, facilitating a variety of imaging positions. Concerning radiation characteristics, the kilovoltage (kV) and milliamperage (mA) were accurately calibrated to achieve optimal image quality with minimized radiation exposure. Moreover, it incorporates advanced safety features such as Automatic Exposure Control and dose reduction algorithms. Standardized anteroposterior and lateral digital radiographs were taken at 4 weeks, 8 weeks, and 12 weeks postoperatively, corresponding to critical stages in the bone regeneration process (Figure 1).

**Figure 2:**
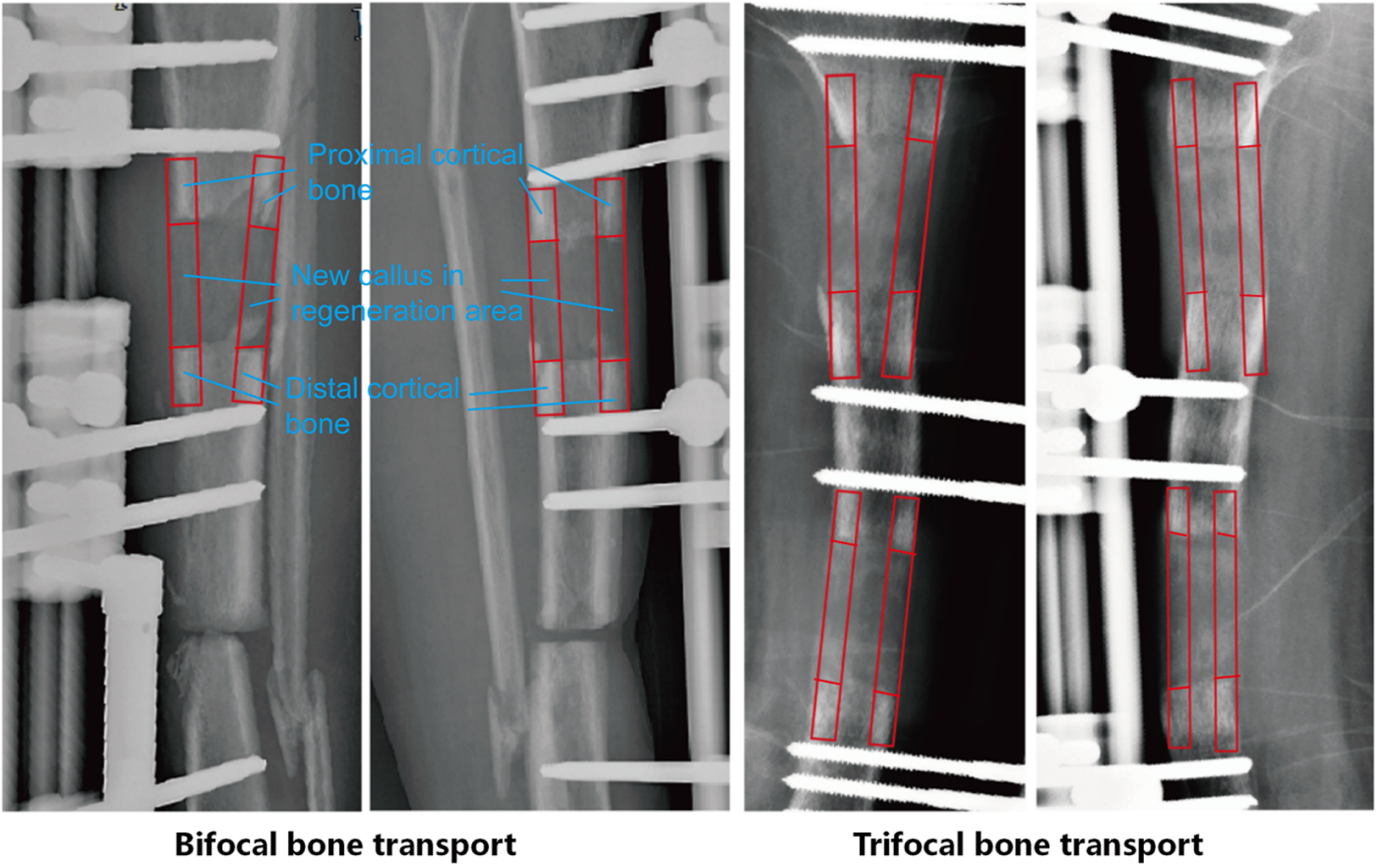
Method for measuring pixel value ratio (PVR) on digital radiographs. Regions of interest (ROIs) are selected in the regenerate bone and adjacent normal bone for PVR calculation.

The pixel values in the study area of the affected limb x-rays were measured, carefully avoiding interference from the external fixator. Measurements were taken from both anteroposterior and lateral views, including the regenerate area, and the proximal and distal normal cortical bone (Figure 2). The PVR was calculated using the following formula: PVR = (Regenerated bone pixel value × 2)/(Proximal normal cortical bone pixel value + Distal normal cortical bone pixel value). We confined the ROIs (Regions of Interest) to the regenerative area as well as the cortical regions of the proximal and distal normal bone. For the regenerative bone, we defined the entire cortical zone of the regenerative segment as the ROI. For the normal bone cortex, we selected the region extending approximately 2 cm from the edge of the bone defect. For each ROI, we calculated the average pixel value and excluded any outliers. The PVR for each of the four cortices (anterior, posterior, medial, and lateral) was calculated, and the mean of these four values was used as the final PVR for each time point. A higher PVR value indicates more advanced bone regeneration, with a value of 1 representing mineralization equivalent to the adjacent normal bone. The mineralization time of the distraction gap was calculated from the point when the transport segment reached its predetermined position. We calculated the average PVR of the 2 regenerate bone sites in TBT group to obtain a comprehensive and representative figure, enabling a direct comparison with the SBBT group. The use of average values helps to reflect the overall situation and reduce potential biases introduced by individual measurement points. Additionally, prior to conducting the multi-group analysis, we performed a variance analysis on the PVR of the two regenerative zones in the TBT group over the first three months. The statistical results indicated no significant difference between the PVR values of the two regenerative zones.

PVR measurements were taken from monthly radiographs, with a specific focus on the 1st, 2nd, and 3rd months post-distraction. Two independent observers, blinded to the clinical outcome, performed all measurements. The average of their measurements was used for analysis. We compared the PVR differences among the SBBT, LBBT, and TBT groups during the first three months of mineralization. This allowed us to analyze the mineralization trends of the distraction gap in bone transport techniques of different lengths and complexities.

### Data collection and outcome measures

Demographic data collected included age, sex, body mass index (BMI), smoking status, alcohol consumption, location of defect (tibia or femur), and length of bone defect. The primary outcome measure was the PVR at 4, 8, and 12 weeks postoperatively. Secondary outcome measures included the healing index (HI), defined as the time to union in months divided by the length of the regenerate in cm, and the external fixation index (EFI), defined as the time the fixator was in place in months divided by the length of the regenerate in cm.

### Statistical analysis

Statistical analysis was performed using GraphPad Prism 9.5.0 (GraphPad Software, San Diego, CA, USA). The Shapiro-Wilk test was used to assess the normality of data distribution. Continuous variables were expressed as mean ± standard deviation. Categorical variables were presented as frequencies and percentages. To compare the PVR values among the three groups (SBBT, LBBT, and TBT), we used one-way ANOVA for each time point (4 weeks, 8 weeks, and 12 weeks). Two-way repeated measures ANOVA was conducted to analyze PVR trends over time among the groups. The HI and EFI were compared among the groups using one-way ANOVA. A *p*-value <0.05 was considered statistically significant.

## Results

### Patient demographics and clinical characteristics

A total of 60 patients (60 limb segments) were included in this study, comprising 22 cases in the Short Bifocal Bone Transport (SBBT) group, 20 cases in the Long Bifocal Bone Transport (LBBT) group, and 18 cases in the Trifocal Bone Transport (TBT) group. The demographic and clinical characteristics of the patients are summarized in Table 1. Male patients predominated in all groups, accounting for 72.7% (16/22) in the SBBT group, 75.0% (15/20) in the LBBT group, and 77.8% (14/18) in the TBT group. This male predominance is consistent with the epidemiology of traumatic bone defects and the general trend in bone transport procedures. The mean age of patients was comparable across the three groups: 34.31 ± 10.70 years in the SBBT group, 32.64 ± 12.66 years in the LBBT group, and 39.45 ± 12.60 years in the TBT group.

**Table 1:** Demographic and clinical characteristics of patients undergoing bone transport.

	SBBT	LBBT	TBT
Sex			
Male	16 (72.7%)	15 (75.0%)	14 (77.8%)
Female	6 (27.3%)	5 (25.0%)	4 (22.2%)
Age (years)	34.31 ± 10.70	32.64 ± 12.66	39.45 ± 12.60
BMI (kg/m^2^)	23.15 ± 3.85	25.18 ± 6.03	25.45 ± 5.07
Smoking (*n* = 12)	5 (41.7%)	3 (25%)	4 (33.3%)
Drinking (*n* = 19)	5 (26.3%)	8 (42.1%)	6 (31.6%)
Surgical site			
Tibia	16 (72.7%)	14 (70.0%)	13 (72.2%)
Femur	6 (27.3%)	6 (30.0%)	5 (27.8%)
Lengthening length	4.06 ± 0.58	7.95 ± 1.00	8.05 ± 1.05
Healing time	5.54 ± 0.52	11.55 ± 1.97	5.91 ± 0.83
External fixed time	7.00 ± 0.82	14.73 ± 2.24	8.18 ± 0.81

SBBT, short bifocal bone transport; LBBT, long bifocal bone transport; TBT, trifocal bone transport.

Regarding the surgical site, the tibia was the predominant location for bone transport in all three groups. Tibial cases accounted for 72.7% (16/22) in the SBBT group, 70.0% (14/20) in the LBBT group, and 72.2% (13/18) in the TBT group. Femoral cases made up the remaining 27.3% (6/22), 30.0% (6/20), and 27.8% (5/18) in the SBBT, LBBT, and TBT groups, respectively. This distribution reflects the higher incidence of tibial defects requiring bone transport in clinical practice. The SBBT group had a mean lengthening of 4.06 ± 0.58 cm, while the LBBT and TBT groups had longer defects with mean lengthenings of 7.95 ± 1.00 cm and 8.05 ± 1.05 cm, respectively.

The healing time and external fixation time also varied among the groups. The SBBT group had the shortest mean healing time (5.54 ± 0.52 months) and external fixation time (7.00 ± 0.82 months). The LBBT group had the longest mean healing time (11.55 ± 1.97 months) and external fixation time (14.73 ± 2.24 months). Interestingly, despite having similar defect lengths to the LBBT group, the TBT group showed intermediate values for both healing time (5.91 ± 0.83 months) and external fixation time (8.18 ± 0.81 months).

### PVR analysis

**Figure 3:**
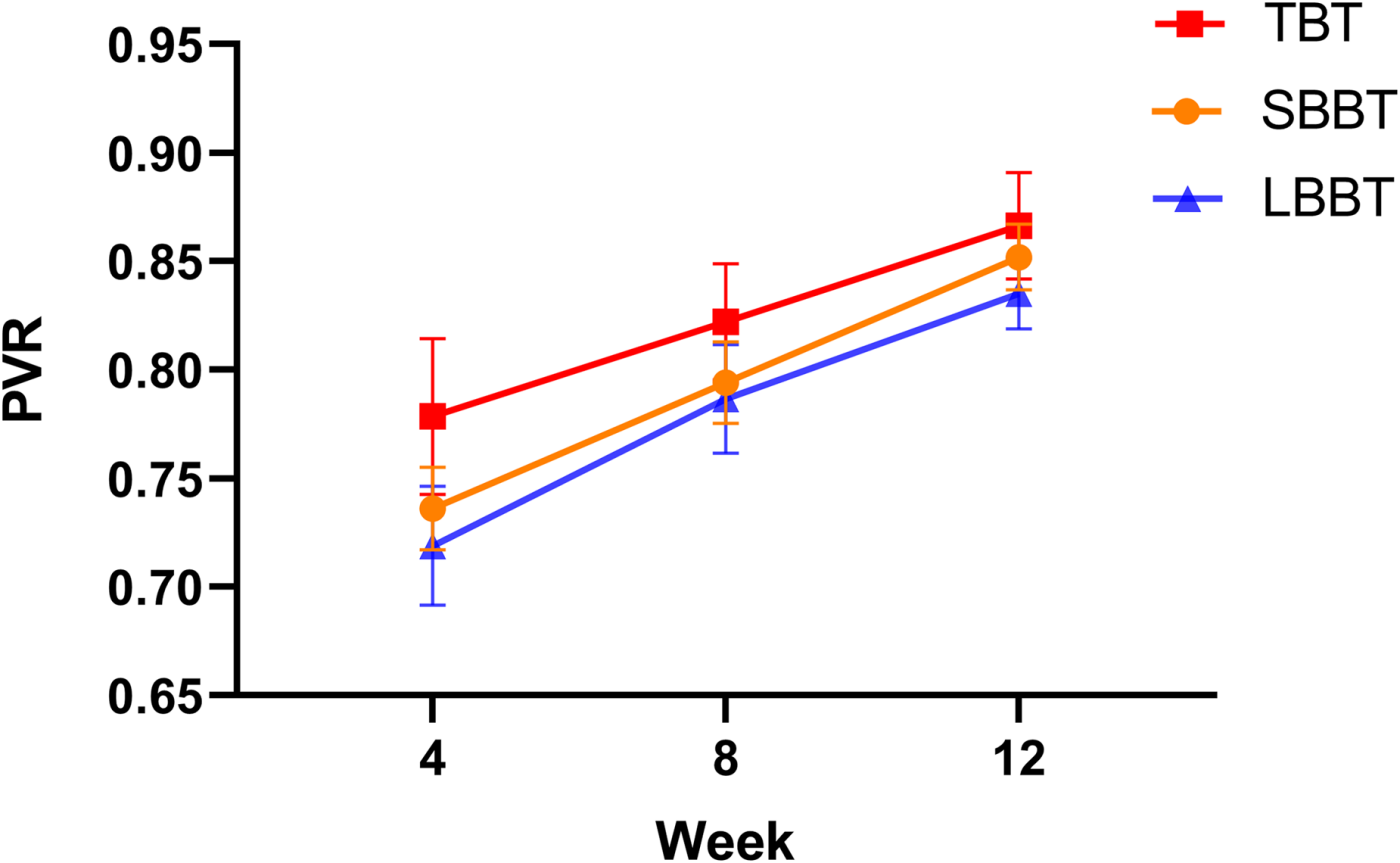
Comparison of mean pixel value ratios (PVR) among short bifocal bone transport (SBBT), long bifocal bone transport (LBBT), and trifocal bone transport (TBT) groups at 4, 8, and 12 weeks post-operation. Error bars represent standard deviation.

The PVR was measured at 4, 8, and 12 weeks post-operatively for all patients. The results for each group are presented in Table 2, and illustrated in Figure 3. We performed a Shapiro-Wilk test to assess the normality of the PVR data. The results indicated that the measured PVR values followed a normal distribution (*P* > 0.05). The maximum PVR value was 0.91, and the minimum PVR value was 0.68.

**Table 2:** Comparison of PVR between SBBT, LBBT and LBBT groups.

	4 weeks		8 weeks		12 weeks	
	PVR value (mean ± SD)	*P* value	PVR value (mean ± SD)	*P* value	PVR value (mean ± SD)	*P* value
SBBT	0.736 ± 0.019	0.127	0.794 ± 0.019	0.756	0.852 ± 0.015	0.137
LBBT	0.719 ± 0.027		0.787 ± 0.025		0.835 ± 0.016	
LBBT	0.719 ± 0.027	<0.001	0.787 ± 0.025	0.008	0.835 ± 0.016	0.023
TBT	0.779 ± 0.036		0.822 ± 0.027		0.866 ± 0.024	
SBBT	0.736 ± 0.019	<0.001	0.794 ± 0.019	0.016	0.852 ± 0.015	0.377
TBT	0.779 ± 0.036		0.822 ± 0.027		0.866 ± 0.024	

SBBT, short bifocal bone transport; LBBT, long bifocal bone transport; TBT, trifocal bone transport.

Comparison between SBBT and LBBT groups (Table 2 and Figure 3): The SBBT group showed consistently higher PVR values compared to the LBBT group throughout the 12-week period. At 4 weeks, the PVR values were 0.736 ± 0.019 for SBBT and 0.719 ± 0.027 for LBBT (*p* = 0.127). At 8 weeks, the values were 0.794 ± 0.019 for SBBT and 0.787 ± 0.025 for LBBT (*p* = 0.756). At 12 weeks, the values were 0.852 ± 0.015 for SBBT and 0.835 ± 0.016 for LBBT (*p* = 0.137). Although the SBBT group consistently showed higher PVR values, these differences were not statistically significant at any time point.

Comparison between LBBT and TBT groups (Table 2 and Figure 3): The TBT group demonstrated significantly higher PVR values compared to the LBBT group at all time points. At 4 weeks, the PVR values were 0.779 ± 0.036 for TBT and 0.719 ± 0.027 for LBBT (*p* < 0.001). At 8 weeks, the values were 0.822 ± 0.027 for TBT and 0.787 ± 0.025 for LBBT (*p* = 0.008). At 12 weeks, the values were 0.866 ± 0.024 for TBT and 0.835 ± 0.016 for LBBT (*p* = 0.023). These results suggest that the trifocal technique may lead to faster bone regeneration compared to the bifocal technique for long bone defects.

Comparison between SBBT and TBT groups (Table 2 and Figure 3): The TBT group showed significantly higher PVR values compared to the SBBT group at 4 and 8 weeks. At 4 weeks, the PVR values were 0.779 ± 0.036 for TBT and 0.736 ± 0.019 for SBBT (*p* < 0.001). At 8 weeks, the values were 0.822 ± 0.027 for TBT and 0.794 ± 0.019 for SBBT (*p* = 0.016). However, the difference was not statistically significant at 12 weeks (TBT: 0.866 ± 0.024, SBBT: 0.852 ± 0.015; *p* = 0.377). This suggests that while the trifocal technique may lead to faster early bone regeneration, the differences become less pronounced over time.

All three groups showed an increasing trend in PVR values over time, indicating progressive mineralization of the regenerate bone. The rate of increase was highest in the TBT group, followed by the SBBT group, and then the LBBT group.

### Healing index and external fixation index

**Figure 4:**
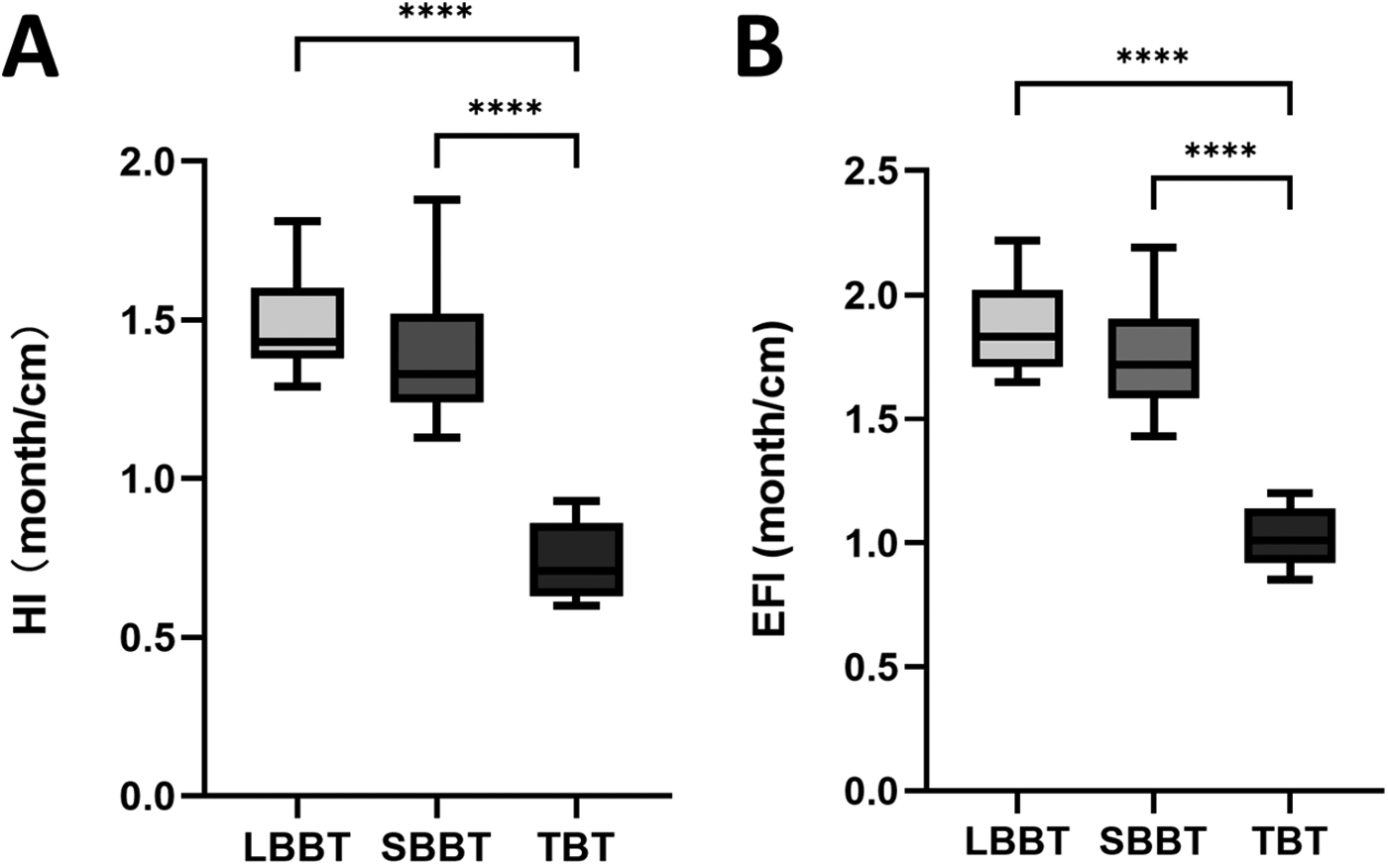
Box plots comparing **(A)** healing index (HI) and **(B)** external fixation index (EFI) among short bifocal bone transport (SBBT), long bifocal bone transport (LBBT), and trifocal bone transport (TBT) groups. The boxes represent the interquartile range, the horizontal line within the box represents the median, and the whiskers extend to the minimum and maximum values.

The healing index (HI) and external fixation index (EFI) for each group are presented in Figure 4, respectively. These indices provide important information about the efficiency of each bone transport technique. The mean HI was significantly lower in the TBT group compared to both the LBBT group and the SBBT group. This indicates that bone defects treated with the bifocal technique heal more quickly per centimeter of lengthening. Similarly, the mean EFI was significantly lower in the TBT group compared to both the LBBT group and the SBBT group. This suggests that patients with bone defects treated with the bifocal technique required less time in the external fixator per centimeter of lengthening. Interestingly, despite the longer bone defects in the TBT group, both the HI and EFI were lower in the TBT group compared to the LBBT group. This finding suggests that the trifocal technique may be more efficient than the bifocal technique for treating longer bone defects, resulting in faster healing and shorter time in the external fixator.

## Discussion

This study presents a comprehensive analysis of bone regeneration rates in different bone transport techniques, utilizing the PVR as a quantitative measure. Our investigation compared short bifocal bone transport (SBBT), long bifocal bone transport (LBBT), and trifocal bone transport (TBT) techniques, focusing on early mineralization rates in different distraction lengths and segments. The results provide valuable insights into the efficacy of these techniques and have significant implications for clinical practice in orthopedic surgery.

One of the key strengths of our study is the use of PVR as an objective, cost-effective method for assessing bone mineralization in the distraction gap. This approach builds upon the work of Vulcano et al. (13), who demonstrated the utility of radiographic pixel density in assessing bone healing during limb lengthening. Our application of PVR to bone transport techniques extends this concept, providing a quantitative comparison of regeneration rates between different techniques and defect lengths. This method offers a more accessible alternative to other quantitative assessment tools such as dual-energy x-ray absorptiometry (DEXA) or quantitative computed tomography (QCT), which, while sensitive, are often impractical for routine clinical use due to cost and radiation exposure concerns (10).

A significant finding of our study is the comparative analysis of short-distance and long-distance bone transport techniques. Although our results indicated slightly faster regeneration rates in short-distance transport, the difference was not statistically significant. This observation aligns with the findings of Sailhan et al. (22), who reported that defect size alone may not be the determining factor in bone regeneration rates. However, when comparing long-distance bifocal transport with trifocal transport of similar lengths, we found significantly higher early PVR growth rates in the trifocal group. This finding supports the work of Borzunov et al. (5), who suggested that multiple osteotomy sites in trifocal transport might enhance biological responses and accelerate bone formation.

Interestingly, when comparing trifocal transport with short bifocal transport of approximately half the length, the trifocal technique showed higher regeneration rates in the early stages, with the difference becoming less pronounced by the third month. This suggests that even individual segments in trifocal transport exhibit faster mineralization rates compared to equivalent-length segments in bifocal transport. This observation extends the findings of Ilizarov (1) and Paley (3), who described the biological advantages of multiple osteotomy sites but did not quantitatively compare regeneration rates between techniques.

Our results indicating higher early bone callus regeneration efficiency and shorter external fixator wear time in trifocal transport have important clinical implications. These findings support the observations of Catagni et al. (23) and Rozbruch et al. (24), who reported favorable outcomes with trifocal transport in complex cases. Our quantitative data provide a stronger evidence base for clinicians to confidently employ trifocal techniques in suitable scenarios, potentially improving early mineralization efficiency and reducing the psychological and physical burden on patients associated with prolonged external fixation.

The correlation between higher PVR values and shorter healing and external fixation indices across all groups is particularly noteworthy. This relationship, consistent with the findings of Song et al. (12), suggests that PVR could serve as a valuable predictor of clinical outcomes in bone transport procedures. The potential of PVR as a prognostic tool aligns with the growing emphasis on objective, quantitative measures in orthopedic decision-making, as highlighted by Babatunde et al. (10).

Our analysis of healing index (HI) and external fixation index (EFI) provided further insights into the clinical efficacy of these techniques. The significantly lower HI and EFI in the SBBT group compared to both LBBT and TBT groups is expected, given the shorter defect lengths. However, the lower HI and EFI in the TBT group compared to the LBBT group, despite similar defect lengths, is particularly intriguing. This finding suggests that the trifocal technique may offer advantages in terms of treatment efficiency for longer bone defects, potentially reducing overall treatment time and associated complications.

The predominance of tibial cases in our study (approximately 70% across all groups) reflects the higher incidence of tibial defects requiring bone transport in clinical practice. This distribution is similar to that reported in other large-scale studies on bone transport by Rohilla et al. (25) and Yin et al. (26). The higher proportion of male patients in our study population is also consistent with the epidemiology of traumatic bone defects and the general trend in bone transport procedures, as observed in the systematic review by Papakostidis et al. (27).

Besides, there might be certain issues with our research design. In clinical practice, the tibia and femur, as the primary long bones of the lower limb, are often studied together in the context of Ilizarov bone transport technique due to their similar biomechanical properties. Based on the similar biomechanical states of these two long bones, we combined them in our study, increasing the sample size and enhancing the persuasiveness of our results. However, we acknowledge that potential differences between the two bones may exist, which could have an impact on our findings. Meanwhile, to avoid interference from different external fixators, we selected patients who wore unilateral external fixators for bone transport as our research subjects. This can effectively reduce the confounding effects of interference factors on our results and improve the accuracy of our research results. However, it also reduces the universality of obtaining the same results in studies using different external fixation devices.

This study has several methodological limitations. Primarily, our comparative approach lacks comprehensiveness. Although the PVR can reflect the degree and rate of bone mineralization, this two-dimensional measurement method cannot comprehensively evaluate bone quality. Considering that TBT, due to its inherent characteristics, may generate bone tissue more rapidly than LBBT, the assessment of bone quality becomes particularly crucial when evaluating different bone transport techniques. We were unable to perform three-dimensional quantitative analysis of callus formation in the bone regeneration area, and we lacked more in-depth bone quality assessment indicators. These limitations restricted our ability to conduct a comprehensive evaluation of regenerated bone quality. Compared to quantitative CT, which can provide more detailed three-dimensional information, the PVR measurement method employed in this study shows significant limitations in assessing bone quality. For future research, we recommend adopting a more comprehensive evaluation approach to thoroughly assess the impact of different bone transport techniques on bone quality.

In addition to the methodological limitations mentioned above, several other limitations of this study should be acknowledged. Firstly, the retrospective nature of the study introduces potential bias in patient selection and data collection, a common limitation in orthopedic research. Secondly, while our sample size was adequate for the primary outcomes, it may limit the power to detect smaller differences between groups or in subgroup analyses. This limitation is particularly relevant when considering the non-significant difference observed between short and long bifocal transport techniques. Thirdly, we did not assess long-term functional outcomes or quality of life measures, which are important considerations in evaluating the overall success of bone transport procedures. The stringent inclusion criteria we employed to minimize confounding factors, while enhancing the internal validity of our results, may limit the generalizability of our findings. This trade-off between internal and external validity is a common challenge in clinical research. Future studies with larger sample sizes and more diverse patient populations could help address this limitation and provide more comprehensive insights into the efficacy of different bone transport techniques across various clinical scenarios.

## Conclusion

This study provides compelling evidence for the efficacy of the trifocal bone transport (TBT) technique in managing bone defects, demonstrating faster bone formation and significantly reduced healing and external fixation times compared to bifocal methods. Through the novel application of the PVR as a quantitative measure of bone regeneration, we have shown that TBT consistently leads to higher PVR values and lower HI and EFI values. These findings suggest that TBT should be considered as a preferred method for treating bone defects, especially when minimizing treatment time is crucial. The PVR method proved to be a reliable, cost-effective tool for assessing bone regeneration, offering the potential for guiding clinical decision-making. Notably, defect length alone may not be the primary factor in determining healing rates in bifocal techniques.

## Data Availability

The original contributions presented in the study are included in the article/Supplementary Material, further inquiries can be directed to the corresponding author.
